# Identification of a potentially avoidable cardiopulmonary resuscitation in hematology and oncology wards

**DOI:** 10.1186/s12904-019-0477-7

**Published:** 2019-11-04

**Authors:** Yeonjoo Choi, Jin Won Kim, Koung Jin Suh, Yoo-Joo Lim, Ji Yun Lee, Beo-Deul Kang, Ji-Won Kim, Se-Hyun Kim, Jeong-Ok Lee, Yu Jung Kim, Keun-Wook Lee, Jee Hyun Kim, Soo-Mee Bang, Jong Seok Lee

**Affiliations:** 1 Division of Hematology and Medical Oncology, Department of Internal Medicine, Seoul National University Bundang Hospital, Seoul National University College of Medicine, 82 Gumi-ro 173 beon-gil, Bundang-gu, Seongnam, 13620 Republic of Korea; 2 Division of Hematology and Medical Oncology, Department of Internal Medicine, Seoul National University Hospital, Seoul National University College of Medicine, Seoul, Republic of Korea; 3 Present Address: Department of Investigational Cancer Therapeutics, The MD Anderson Cancer Center, Houston, TX USA; 4 Present Address: CHA Bundang Medical Center, CHA University, Seongnam, Republic of Korea

**Keywords:** Cardiopulmonary arrest, Resuscitation, Cancer, Potentially avoidable, Prognosis

## Abstract

**Background:**

In-hospital cardiopulmonary resuscitation (CPR) is one of undesirable situations. We tried to identify and characterize a potentially avoidable CPR in cancer patients who were hospitalized in hematology and oncology wards.

**Methods:**

A potentially avoidable CPR was determined based on chemotherapy setting, disease status and clinical situation at the time when a cardiopulmonary arrest occurred, by using a consensus-driven medical records review of two physicians.

**Results:**

One hundred thirty-seven patients among 12,437 patients hospitalized at hematology and oncology wards between March 2003 and June 2015 (1.1%) underwent a CPR. Eighty-eight patients (64.2%) were men. The majority of patients with a CPR had lung cancer (41, 29.9%), hematologic malignancy (24, 17.5%), stomach cancer (23, 16.8%) or lymphoma (20, 14.6%). A potentially avoidable CPR was identified in 51 patients (37.2%). In a multivariate analysis, advanced diseases and certain tumor types (e.g., lung cancer, lymphoma) were significant risk factors for a potentially avoidable CPR. Of patients who received a potentially avoidable CPR, 29 patients (56.9%) did not have a do-not-resuscitate documentation. A first return of spontaneous circulation rate (ROSC) and in-hospital survival rate (IHSR) were much lower in patients with a potentially avoidable CPR than those with a CPR that was not avoidable (ROSC: 39.2% vs 53.5%, *P* = 0.106; IHSR: 2.0% vs 12.8%, *P* = 0.032, respectively).

**Conclusions:**

A potentially avoidable CPR was common at hematology and oncology wards. A potentially avoidable CPR frequently occurred in advanced diseases and certain tumor types. Furthermore, cancer patients who received a potentially avoidable CPR showed the worse prognosis.

## Background

In-hospital cardiopulmonary arrest is one of the most undesirable events in caring for patients. Cancer patient is one of the largest in-hospital cardiopulmonary arrest groups. However, most of them do not experience the return of systemic circulation (ROSC) after a cardiopulmonary resuscitation (CPR). Even among those who achieved ROSC, only few are discharged from hospital. In one study, 16 of 73 cancer patients (22%) who had sudden, unanticipated cardiac arrest survived to be discharged from a hospital, but none (0 of 171) of patients who experienced an anticipated cardiac arrest survived [1]. Another meta-analysis revealed that the overall survival to discharge rate in cancer patients was only 6.2% [2]. In the view of these studies, a CPR seems to be less meaningful to resuscitate cancer patients [3]. Furthermore, aggressiveness of cancer care techniques near the end of life, such as a CPR, can deteriorate patients’ quality of life and increase costs related to care [4, 5]. Recently, patients who underwent an early palliative care including advanced care planning for do-not-resuscitate (DNR) showed an improved survival outcome compared with those who experienced a standard care [6].

Clinical outcomes in general CPR population have been improved through advanced knowledge including basic CPR before defibrillation, the use of automated external defibrillators with easy access, new resuscitation algorithms including cardiac compression rates, ventilation rates and volumes, and hypothermia therapy [7–9]. End-of-life and advanced care planning also may have contributed to improved outcomes of a CPR in the general population by allowing terminally ill patients to choose a DNR, which were effectively decreasing the number of patients that would have worse outcomes [10]. In metastatic cancer patients, it was reported that overall survival to discharge has been improved from 5.6 to 7.8% in recent years, reflecting more selective use of a CPR in cancer patients, with the sickest patients deselected [2]. Through a focus on palliative care for cancer patients and an incorporation of patient goals of care in deciding therapeutic interventions, CPR might be applied more selectively, resulting in higher rates of ROSC and longer survival after CPR. Therefore, selecting out patients who are likely to have the worst prognosis before CPR and excluding them from using a CPR should ultimately improve the total outcome. The identification of a potentially avoidable CPR is an important issue in the palliative care of the cancer.

In this study, we tried to identify the incidence and characteristics of a potentially avoidable CPR in cancer patients who were hospitalized in hematology and oncology wards by using a consensus-driven peer review process. We also sought to evaluate ways in which a potentially avoidable CPR could not be conducted.

## Methods

### Patient selection

All patients were admitted to hematology and oncology wards. All patients were diagnosed with any kind of cancer. Among all patients who experienced cardiopulmonary arrest events at hematology and oncology wards of the Seoul National University Bundang Hospital between March 2003 and June 2015, we selected consecutive series of patients who received a CPR. All patients experienced a cardiopulmonary arrest and were given a CPR, including the cardiac massage, the intubation, the direct current cardioversion or the intensive care unit treatment.

### Identification of a potentially avoidable CPR

Based on chemotherapy setting, disease status and clinical situation at the time when the arrest event occurred, whether that event was potentially avoidable or not was determined [11]. The potentially avoidable CPR was defined as a CPR for cardiopulmonary arrest events in patients who had no further chemotherapy plan, the hospice care, or expected worse clinical courses with the irreversible prognosis. For example, a CPR in patients who were waiting to transfer to hospice care unit or were given just palliative therapy with no further chemotherapy plan was classified into a potentially avoidable CPR. A CPR from contrast-induced anaphylaxis or during chemotherapy infusion was classified into a CPR that was not avoidable. A physician entered all clinical characteristics into a clinical research form and reviewed all patients. Another physician independently reviewed the patients’ information in the access record to determine whether that event was potentially avoidable or not. If there was a disagreement between two independent physicians, the classification was confirmed through further discussion case by case.

### Data collection

Some specific queries were used to gather the information about cardiopulmonary arrest events in patients’ electric medical records. These queries included “Medical Providers cannot check pulse rate.”, “Patients have no self-respiration.”, “Cardio-pulmonary arrest happens.”, and “Doctors provide resuscitation with cardiac massage and/or intubation.”. For patients whose date of death could not be verified through the electronic medical record, death statistics were obtained from the Ministry of Public Administration and Security in Korea.

### Statistical analysis

Categorical variables were summarized using frequencies and percentages, whereas the continuous variables were summarized using descriptive statistics such as the median and 25–75% range. Differences of clinical parameters between two groups were assessed using a chi-squared test. A multivariate analysis of factors associated with a potentially avoidable CPR was conducted using Cox’s proportional hazards model. *P*-values less than 0.05 were considered statistically significant. The statistical analysis was performed using SPSS 19.0 K for Windows (SPSS Inc. Chicago, IL, USA).

### Ethics statement

This study was approved by the Seoul National University Bundang Hospital institutional review board (IRB No: B-1507/306–104). Requirement for informed consent was waived and data collection was conducted under accordance with World Medical Association’s Declaration of Helsinki.

## Results

### Patients’ characteristics

There were 12,437 cancer patients hospitalized in the hematology and oncology wards at Seoul National University Bundang Hospital between March 2003 and June 2015. Among these patients, 137 patients (1.1%) underwent a CPR for cardiopulmonary arrest events. The mean age of patients receiving a CPR was 67.5 years. Most patients were older than 60 years (Table 1). Twenty-four patients (17.5%) were over 80 years old. Eighty-eight patients were male (64.2%). The majority of patients who underwent a CPR had lung cancer (41, 29.9%), hematologic malignancy (24, 17.5%), stomach cancer (23, 16.8%) or lymphoma (20, 14.6%). In terms of reasons of hospitalization, 70 patients (51.1%) were admitted for the supportive care due to cancer symptoms, and 51 patients (37.2%) had a planned hospitalization such as a scheduled chemotherapy or another procedure. The clinical settings of cancer treatments in patients with a CPR were curative/adjuvant, no prior chemotherapy, and the palliative in 24 (17.5%), 30 (21.9%), and 83 (60.6%) patients, respectively. The median interval from the last chemotherapy to a CPR was 31.0 days.

**Table 1:** Baseline characteristics

Variables		Potentially avoidable		
	All, *N* = 137, (%)	Yes, *N* = 51 (%)	No, *N* = 86 (%)	*p* value
Age group (year)				0.969
-60	41 (29.9)	15 (36.6)	26 (63.4)	
61–70	30 (21.9)	11 (36.7)	19 (63.3)	
71–80	42 (30.7)	16 (35.7)	26 (64.3)	
81+	24 (17.5)	10 (41.7)	14 (58.3)	
Sex				0.409
Male	88 (64.2)	35 (39.8)	53 (60.2)	
Female	49 (35.8)	16 (32.7)	33 (67.3)	
Cancer type				0.021
Lung cancer	41 (29.9)	20 (48.8)	21 (51.2)	
Hematologic malignancy	24 (17.5)	2 (8.3)	22 (91.7)	
Stomach cancer	23 (16.8)	9 (39.1)	14 (60.9)	
Lymphoma	20 (14.6)	6 (30.0)	14 (70.0)	
Oesophageal cancer	5 (3.6)	2 (40.0)	3 (60.0)	
Pancreas-biliary cancer	5 (3.6)	2 (40.0)	3 (60.0)	
Breast cancer	5 (3.6)	4 (80.0)	1 (20.0)	
Colorectal cancer	4 (2.9)	3 (75.0)	1 (25.0)	
Other	10 (7.3)	3 (30.0)	7 (70.0)	
Reasons of hospitalization				0.207
Cancer symptom	70 (51.1)	31 (44.3)	39 (55.7)	
Planned	51 (37.2)	17 (33.3)	34 (66.7)	
Treatment complication	9 (6.6)	2 (22.2)	7 (77.8)	
Non-cancer medical condition	7 (5.1)	1 (14.3)	6 (85.7)	
Clinical setting at CPR				< 0.001^a^
Curative/adjuvant	24 (17.5)	3 (12.5)	21 (87.5)	
No prior chemotherapy	30 (21.9)	8 (26.7)	22 (73.3)	
1st palliative	29 (21.2)	10 (34.5)	19 (65.6)	
2nd palliative	25 (18.2)	10 (40.0)	15 (60.0)	
3rd palliative or more	29 (21.2)	20 (69.0)	9 (31.0)	
Interval from last chemotherapy (median, 25–75%, days)	31.0 (8.5–56.0)	37.0 (13.0–63.0)	29.5 (8.0–52.0)	

*CPR* cardiopulmonary resuscitation

^a^ Linear-by-linear association

A potentially avoidable CPR was applied in 51 patients (37.2%). There was no significant difference between patients with a potentially avoidable CPR and those with a CPR that was not avoidable according to age, sex and reasons of hospitalization (*p* = 0.969, *p* = 0.409, *p* = 0.207, respectively). However, there was a significant difference according to tumor types and clinical settings (*p* = 0.021 and *p* < 0.001, respectively). Patients with breast cancer, colorectal cancer and lung cancer received a potentially avoidable CPR more frequently (*p* = 0.021). In more advanced clinical settings, a potentially avoidable CPR was also identified more frequently than in less-advanced settings (*p* < 0.001).

### Causes of cardio-pulmonary arrest

The causes leading to a CPR were various (Table 2). Irreversible cancer progression-related symptoms (25.5%) were the most common causes for a CPR, followed by chemotherapy-induced infection (16.8%) and cancer bleeding (16.1%). Some patients experienced cardiopulmonary arrest events related to a primary medical problem such as a heart disease and procedure- or treatment- related complications. The common causes of a potentially avoidable CPR were also cancer progression-related symptoms (58.9%). However, in patients with a CPR that was not avoidable, chemotherapy-induced infection and cancer bleeding were common (24.4 and 18.6%, respectively). There was a statistically significant difference between potentially avoidable and unavoidable group according to the cause (*p* < 0.001).

**Table 2:** Causes of cardiopulmonary arrest

		Potentially avoidable *	
	All, N = 137, (%)	Yes, N = 51 (%)	No, N = 86 (%)
Irreversible Cancer progression-related symptoms	35 (25.5)	30 (58.9)	5 (5.8)
Infection, chemotherapy-induced	23 (16.8)	2 (3.9)	21 (24.4)
Cancer bleeding	22 (16.1)	6 (11.8)	16 (18.6)
Primary respiratory failure (ex: asphyxia, aspiration etc.)	14 (10.2)	6 (11.8)	8 (9.3)
Infection, not chemotherapy-induced	13 (9.5)	4 (7.9)	9 (10.5)
Heart disease	10 (7.3)	1 (1.9)	9 (10.5)
Respiratory failure due to other disease (e.g.: acute kidney injury due to chemotherapy)	5 (3.6)	1 (1.9)	4 (4.6)
Procedure/treatment-related complication, except infection (ex: anaphylaxis)	5 (3.6)	0 (0)	5 (5.8)
Others and unknown	10 (7.3)	1 (1.9)	9 (10.5)

*CPR* cardiopulmonary resuscitation, * *P* value < 0.001

### Factors associated with a potentially avoidable CPR

In a multivariate analysis, clinical setting at the time when the CPR was conducted and primary tumor types were significantly associated with a risk of conducting a potentially avoidable CPR (Table 3). A potentially avoidable CPR was more frequently identified in patients who underwent palliative chemotherapy, compared to patients who underwent curative/adjuvant therapy (odds ratio [OR] = 20.19, *p* = 0.004). Classified by primary tumor type, a potentially avoidable CPR was also more frequently applied in patients with lung cancer, lymphoma, colorectal cancer or breast cancer than in patients with hematologic malignancies (OR = 6.53, *p* = 0.033; OR = 11.60, *p* = 0.018; OR = 18.09, *p* = 0.050; and OR = 16.42, *p* = 0.052, respectively). However, age was not a risk factor for a potentially avoidable CPR.

**Table 3:** Multivariate analysis of risk factors associated with a potentially avoidable CPR

	Odds ratio (95% CI)	*p* value
Age		
-60	1	
61–70	1.25 (0.37–4.26)	0.720
71–80	1.22 (0.40–3.66)	0.729
81+	2.16 (0.58–8.03)	0.252
Clinical setting at CPR		
Curative/adjuvant	1	
No prior chemotherapy	1.96 (0.31–12.46)	0.478
1st palliative	3.96 (0.58–27.05)	0.161
2nd palliative	4.84 (0.66–35.82)	0.123
3rd palliative	20.19 (2.54–160.18)	0.004
Primary disease		
Hematologic malignancy	1	
Colorectal cancer	18.09 (1.00–329.20)	0.050
Oesophageal cancer	8.11 (0.66–100.37)	0.103
Lung cancer	6.53 (1.17–36.46)	0.033
Lymphoma	11.60 (1.52–88.77)	0.018
Pancreato-biliary cancer	8.58 (0.71–103.81)	0.091
Stomach cancer	4.21 (0.69–25.72)	0.120
Breast cancer	16.42 (0.98–275.38)	0.052
Other disease	3.54 (0.41–30.49)	0.249

*CPR* cardiopulmonary resuscitation, *CI* confidence interval

### Assumed reasons for conducting a potentially avoidable CPR

Of patients with a potentially avoidable CPR, 29 (56.9%) patients did not provide an informed DNR documentation before a cardiopulmonary arrest and even remaining 22 patients (43.1%) were informed of clinical course and the DNR (Table 4). Among these 22 patients provided a DNR documentation, 10 (45.5%) needed more time to discuss the DNR because they had not made a final decision. In 9 patients (40.9%), a CPR was requested by patient’s family members, even though physicians discussed this option with them and explained that it was not necessary. Three events (13.6%) occurred just before family members arrived.

**Table 4:** Assumed reasons for conducting a potentially avoidable CPR

	Number	Percent
Previous discussion for DNR	22	43.1^a^
Needed more time to discuss DNR	10	45.5^b^
Family members’ request	9	40.9^b^
Discussed but unexpected event or until family arriving	3	13.6^b^
No informed documentation before the events	29	56.9^a^

*CPR* cardiopulmonary resuscitation, *DNR* do-not-resuscitate

^a^Of patients with the potentially avoidable CPR, ^b^Of patients who provided DNR documentation

### Outcome and prognosis of a CPR

**Fig. 1:**
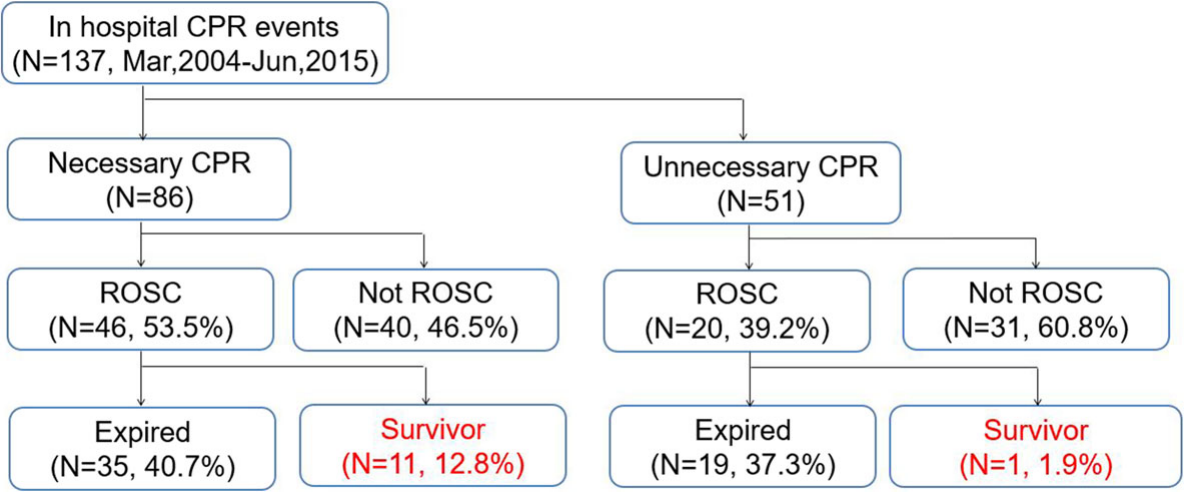
Outcomes of resuscitation attempts. CPR, cardiopulmonary resuscitation; ROSC, return of spontaneous circulation

Of 137 patients who received a CPR, ROSC was achieved in 66 patients (48.2%) (Fig. 1). Of patients with a CPR that was not avoidable, 46 patients (53.5%) achieved ROSC, but only 20 (39.2%) did in patients with a potentially avoidable CPR. Although there was not statistically significant, this ROSC rate in patients with a potentially avoidable CPR was lower than in patients with a CPR that was not avoidable (*P* = 0.106). After ROSC, compared with 11 (23.9%) of patients with a CPR that was not avoidable, only one (5.0%) of patients with a potentially avoidable CPR survived through to discharge from a hospital. Although there was not statistically significant (*P* = 0.088), in-hospital survival rate (IHSR) after ROSC was also much lower in patients with a potentially avoidable CPR than in patients with a CPR that was not avoidable. Following these survivors after a discharge, 5 of patients with a CPR that was not avoidable died within two weeks and one patient with a potentially avoidable CPR died within 1 day after a discharge. The other patients showed long-term survival for more than 2 months, and three patients lived for more than 1 year. Finally, overall IHSR were 2.0 and 12.8% in patients with a potentially avoidable CPR and patients with a CPR that was not avoidable, respectively. This difference of overall IHSR was statistical significant (*P* = 0.032). Detailed characteristics of the survivors are described in Table 5.

**Table 5:** Characteristics of survivors

No.	Age	Sex	Primary disease	Clinical setting at CPR	Cause of arrest	Survival time from discharge (days)
1	60 (±5)	M	Acute leukaemia	No prior chemotherapy	Asphyxia	229
2	70 (±5)	F	Acute leukaemia	Curative	Cancer bleeding	2
3	75 (±5)	M	Stomach cancer	More than 3rd line	Procedure-related complication	135
4	65 (±5)	M	Colon cancer	More than 3rd line	Infection related chemotherapy	1568^a^
5	70 (±5)	F	Stomach cancer	1st palliative	Aspiration	66
6	70 (±5)	M	Lung cancer	More than 3rd line	Cancer related hypercalcaemia	12
7	70 (±5)	F	CML	Curative	Dye hypersensitivity	2225^a^
8	85 (±5)	F	Lung cancer	1st palliative	Infection related chemotherapy	2
9	80 (±5)	M	Multiple myeloma	1st palliative	Ventricular tachycardia	380
10	60 (±5)	M	Pancreatic cancer	1st palliative	Infection-related chemotherapy	12
11	70 (±5)	F	Acute leukaemia	Curative	Infection-related chemotherapy	9
12	60 (±5)	F	Colon	More than 3rd line	Ventricular fibrillation	2

^a^ Alive. *CPR* cardiopulmonary resuscitation, *CML* chronic myelogenous leukaemia

## Discussion

In this present study, 137 patients (1.1%) of all hospitalized patients during the study period received a CPR. Among these 137 patients, a potentially avoidable CPR was identified in 37.2% patients. A potentially avoidable CPR was associated with certain cancer types and clinical settings at the time with a CPR. Physicians should pay more attention to these patients to prevent a potentially avoidable CPR.

In the present study, 66 patients (48.2%) achieved ROSC and 12 (8.8%) patients survived through to discharge. These results is in accordance with a previous meta-analysis report of 1707 cancer patients with a CPR, in which ROSC was 45.4 and 12.6% of ROSC was alive to hospital discharge [2]. Furthermore, in the present study, patients with a potentially avoidable CPR showed much worse prognosis than those with a CPR that was not avoidable (ROSC: 39.2% vs. 53.5%, respectively; overall alive to discharge: 2.0% vs. 12.8%, respectively). Even one patient who survived to discharge in a potentially avoidable CPR died within 1 day after discharge. These findings are also similar to the previous study, in which 22% patients who had sudden, unanticipated cardiac arrest survived to be discharged from the hospital, but none of the patients who experienced an anticipated cardiac arrest survived [1]. Therefore, it is important to identify a potentially avoidable CPR to effectively apply a CPR and improve the outcomes of a CPR in cancer patients.

A potentially avoidable CPR was identified according to chemotherapy setting, disease status and clinical situation at the time when cardiopulmonary arrest occurred in the present study. Considering different outcomes of a CPR between patients with a potentially avoidable CPR and those with a CPR that was not avoidable, the identification of a potentially avoidable CPR using these conditions may be useful and valuable to decide whether to apply a CPR or not in the clinical practice. In a potentially avoidable CPR, most causes of cardiopulmonary arrest were irreversible cancer progression related. In contrast, the causes leading to a CPR that was not avoidable were treatment-related or reversible cancer-related symptoms such as cancer bleeding and infection. The reversibility of causes was the most important factor in a classification of a potentially avoidable CPR and a CPR that was not avoidable. In a multivariate analysis, certain cancer types such as lung cancer and lymphoma were a risk factor for potentially avoidable CPR. These findings were not fully explained, but could be attributed to more aggressiveness of physician for cancer treatment until terminal status due to many treatment options and long disease course in these cancer types.

In 29 patients (56.9%) of patients with a potentially avoidable CPR, there was no documented discussion of a CPR or DNR. The other patients who underwent a potentially avoidable CPR did not fully discuss these items before events occurred. Recently, early palliative care, including advanced care planning, has been shown to lead to better survival and quality of life in palliative cancer patients [6]. Perception of patients and their family members for diagnosis and prognosis is significantly associated with patients’ resuscitation preferences [11–13]. Therefore, the first step to decrease a potentially avoidable CPR should be to discuss disease status and advanced care planning with patients or their family members before the cardiopulmonary arrest event. In fact, there had been little discussion about DNR and hospice care due to prevailing social moods in Korea [14, 15]. However, this social mood has been changed in the past 10 years, and nowadays many patients talk to their doctors to make a decision about their care [16]. In this study, the number of potentially avoidable CPR had an increasing trend until 2011. The events happened the most frequently by 9 cases in 2011. Six cases occurred in each 2006, 2007, 2009, and 2010. There were 5 cases in 2004, 2 in 2008, and 1 in 2005. However, the Korean Ministry of Health and Welfare with government started discussing this social issue in 2012. The number of events got decreased from 2012 of 4 cases, and there was one case in 2013. Seoul National University Bundang Hospital set up a new department for palliative care and started inpatient and outpatient service in 2015. It helped medical providers to prevent unwanted arrest events in hospital. As a result, there was only one case in 2015.

As results, the cases of a potentially avoidable CPR could have decreased, but some families still want resuscitation despite its irreversibility. The more time and the information about a disease course should be provided to these patients and their family members [17, 18]. As one of tools to provide more information and enlighten patients and their family members for a CPR decision making, video decision support tool has been suggested. In a randomized controlled trial, patients with a 3 min video for describing CPR and the chance of CPR success had better CPR knowledge and were more likely to choose DNR compared with a verbal narrative group [19].

Our study had some limitations. First, we reviewed medical records retrospectively, and it was possible that these records did not reflect the patients’ situation accurately and objectively. Second, other physician could have different perspectives to determine a potentially avoidable CPR from this study. In this study, two independent physicians were incorporated to identify a potentially avoidable CPR. If there was any discrepancy between two physicians, we tried to objectively determine it through consensus-driven discussion. Third, in terms of survivors after CPR, quality of life is an important issue. However, we followed just survival and analysed it. We did not address quality of life for survivors. Finally, because we only studied localized, in-hospital CPR, patients without-hospital cardiopulmonary arrest were excluded.

## Conclusions

In conclusion, of total CPR cases with cancer, a potentially avoidable CPR was identified in 37.2% patients. A potentially avoidable CPR frequently occurred in advanced diseases and certain tumor types, such as lung cancer and lymphoma. This information should be used to prevent a potentially avoidable CPR. Furthermore, cancer patients who received a potentially avoidable CPR showed worse outcomes. Therefore, an effort to identify a potentially avoidable CPR should be needed to effectively apply a CPR and improve outcomes of a CPR in cancer patients.

## Data Availability

The datasets used and/or analyzed during the current study are available from the corresponding author on reasonable request.

## Abbreviations

CPR: Cardiopulmonary resuscitation
DNR: Do-not-resuscitate
IHSR: In-hospital survival rate
ROSC: Return of spontaneous circulation rate
